# Delayed Primary and Specialty Care: The Coronavirus Disease–2019 Pandemic Second Wave

**DOI:** 10.1017/dmp.2020.148

**Published:** 2020-05-07

**Authors:** Eric Weinstein, Luca Ragazzoni, Frederick Burkle, Mea Allen, David Hogan, Francesco Della Corte

**Affiliations:** 1 Research Center in Emergency and Disaster Medicine (CRIMEDIM), Università degli Studi del Piemonte Orientale, Novara Italy; 2 Harvard Humanitarian Initiative, Harvard University and T H Chan School of Public Health, Boston, Massachusetts; 3 Woodrow Wilson International Center for Scholars, Washington, DC; 4 Del Valle Institute for Emergency Preparedness, Office of Public Health Preparedness, Boston Public Health Commission, Boston, Massachusetts; 5 Educational Development, TeamHealth Academic Consortium, Moore, Oklahoma

**Keywords:** COVID-19, disaster recovery, incident command system, primary care, surge capacity

## Abstract

Time is of the essence to continue the pandemic disaster cycle with a comprehensive post-COVID-19 health care delivery system RECOVERY analysis, plan and operation at the local, regional and state level.The second wave of COVID-19 pandemic response are not the ripples of acute COVID-19 patient clusters that will persist until a vaccine strategy is designed and implemented to effect herd immunity. The COVID-19 second wave are the patients that have had their primary and specialty care delayed. This exponential wave of patients requires prompt health care delivery system planning and response.

After a sudden onset mass casualty incident, the destruction and degradation of the health care delivery system creates longitudinal problems until the public infrastructure gradually returns: electricity becomes reliable, water is fit to drink, cell towers are resurrected, and the roads are opened. Buildings are inspected, adapted to and eventually returned to duty, or alternative care sites are identified and fitted to accommodate staff, stuff, and the systems to deliver care. Outside staffing resources are welcomed by the community to augment the familiar faces. Supply lines restore the last link in the chain of health care. Recovery of the health care delivery system, this fourth phase of a sudden onset disaster cycle, has been planned and exercised, even studied after hurricanes, floods, and typhoons. There is no model of health care delivery system recovery after a pandemic.

The impact of the coronavirus disease–2019 (COVID-19) pandemic response phase has already exceeded the capacity and capabilities of most city, regional, state, and national health care delivery systems. There are clinical, non-clinical, and support staff who are assigned to attend non-COVID-19 patients in the acute care setting, but the overwhelming majority of staff has been consumed in acute care hospitals for the COVID-19 response. Some have fallen ill or have had to be quarantined. The breadth of the toll of a prolonged acute COVID-19 pandemic response on the well-being and mental health of staff has yet to be appreciated and addressed. Non-acute care staff or acute care staff who typically do not attend to pneumonia or sepsis patients with skills that can be adapted to the acute COVID-19 care environment have been redeployed from their usual practice settings with just-in-time education.

Throughout the world, government restrictions have canceled, postponed, or limited by priority primary and specialty care visits. Additionally, the fear of acquiring the severe acute respiratory syndrome, novel coronavirus 2 (SARS-nCoV-2) has kept people away from their health care providers.^1^ In the United States, this has reduced health care system revenue, specifically the decrease in elective surgical, endoscopy, and other procedures. Across the United States and in most other countries, there has been a marked decrease in emergency department visits and subsequent admissions that have decreased hospitals’ bed census to open space for COVID-19 patients during this slow developing pandemic mass casualty incident; this has also decreased revenue.^2^ Hospitals have been adhering to the mass casualty surge capacity theory to limit or postpone elective surgery and imaging services and to also free in-patient beds (and imaging services) for the acute COVID-19 response; this has also decreased revenue. In the United States, there is a growing number of health care delivery systems that have furloughed clinical, non-clinical, and support staff deemed not essential to the acute COVID-19 response to reduce the impact of projected future revenue loss.^3^ The staff affected by this health care delivery service disruption remain available to return to their duty stations.

Non-acute COVID-19 staff redeployed will likely remain redeployed as the duration of acute care can range from days to weeks. Post-acute care COVID-19 patients who are too weak to go home or require oxygen, who have not been cleared of the SARS-nCoV-2 virus, have few options to transfer out of an acute care bed. They may need physical therapy and pulmonary rehabilitation. The spoke-and-hub system is functioning in most health care delivery systems, with those acute COVID-19 patients who require a ventilator and other intensive care unit management being transferred to tertiary level acute care or alternative site hospitals. The reverse triage process is less functional in most health care delivery systems where the post-acute care COVID-19 patients are transferred to acute care hospitals that can manage these patients. Staff and stuff will still have to be devoted to these facilities.

Stuff has been consumed at an alarming rate with materials taken from non-acute clinical settings; their storage and their contracted-to-be-delivered materials have been redistributed to the acute COVID-19 hospitals. Supply lines have been disrupted from raw materials to finished goods with industries retooled to devote their output for the acute COVID-19 response. Alternative COVID-19 care sites have been adapted to function for an extended period of time in non-acute care structures (wards, clinics, offices), displacing this clinical office and treatment space for an unknown period of time. The collective attention of health care administrators has been focused on their systems’ acute pandemic response.

The acute COVID-19 pandemic will persist until a vaccine has been created, properly tested without political exigency, and administered to create sufficient population immunity. There will be an untold and unpredictable number of short-term rises in patients with an acute COVID-19 infection. These will be mere ripples and will not approximate the Spanish flu of 1918–1919 or modeled COVID-19 projections due to the population’s compliance with masking, social distancing, and hygiene. The second wave of the COVID-19 pandemic is coming.

The second wave will comprise patients with chronic illness who have patiently waited to reschedule or schedule their primary or specialty care appointment to refill medications, obtain durable medical equipment, and undergo surveillance laboratory or imaging studies to gauge effectiveness of titrated therapy. This will include patients who have waited and have symptoms, signs, and other indicators of a serious illness that requires a timely diagnosis to maximize effective treatment. This will include the patients with mental illness or substance abuse who have had their outpatient treatment routines disrupted while doing their best to accept their individual, family, and community stress. Eventually, these patients will need care and, like the COVID-19 response, there will be an exponential curve of presentations and consequences of delayed care: the second wave. Also, there will be post-acute COVID-19 patients with an unknown post-acute pathophysiology who will require longitudinal care; some may require supplemental oxygen for extended periods with a new or adapted pulmonary rehabilitation.

The current acute COVID-19 health care delivery incident command system will remain in place, albeit contracting and expanding in relationship to the ongoing local response. Guided by the Runkle et al.^4^ study from 2012, a secondary surge after a pandemic has parallels to that after a sudden onset disaster. The time is now to stand up a RECOVERY health care delivery system. This would include the post-acute COVID-19 patients who require ongoing care and the primary and specialty health care delivery system that has been canceled, postponed, or limited. The second wave of the COVID-19 pandemic will include the patients who have had their primary and specialty care delayed. There are published post-acute sudden onset disaster response health care system studies after hurricanes, typhoons, floods, tornadoes, a superstorm, tsunami, and nuclear reactor meltdown and forest fires. The delays in patients’ care were measured in days or weeks, not months after the ongoing, acute COVID-19 pandemic response. There are no post-pandemic health care delivery system recovery studies, nor any theoretical studies of a post-pandemic health care delivery system recovery operation. The U.S. Federal Emergency Management Agency (FEMA) National Disaster Recovery Framework^5^ is applicable for most counties post-sudden onset disaster. Adapting these health and social services is a critical strategy to the post-acute COVID-19 environment:

- Identify acute COVID-19 staff: logistics, clinical, support, and administrative and determine how long they will be required to maintain their acute COVID-19 duty.
- Identify non-acute COVID-19 staff: idled, furloughed, or redeployed to the acute-COVID-19 care environment. Determine the time frame to develop a training program for this staff unaccustomed to operate in their duty stations using appropriate personal protective equipment (PPE). When developing this program, consider that the staff may be deployed to a non-acute COVID-19 alternative care site and may have to use alternative or adaptive materials.
- Inventory non-acute COVID-19 stuff that has been consumed, redeployed, or redirected to the acute COVID-19 response to determine a timeline for replacement or how to adapt available materials to be viable in a non-acute COVID-19 primary and specialty care environment.
- Identify the timeline to return non-COVID-19 clinical and other structures or spaces to their prior functional status.
- Identify non-acute COVID-19 alternative primary and specialty care structures (wards, clinics, offices, outpatient surgical and imaging) and spaces (examination and treatment rooms).
- Complete an assessment of primary and specialty care within that specific health care delivery system and prioritize these needs based on the input and participation from the primary and specialty care providers, as well as patients and families in the health care delivery system recovery planning process; and develop a comprehensive timeline for the most-to-least essential services that include consideration of available staff, stuff, and structures to commence the delivery of this care as soon as possible to reduce the untoward effects of delayed care.
- Involve the non-acute COVID-19 staff who are waiting to return to their duty station to learn the evolving recommendations as directed by the recovery incident command system in the process to adapt their duty station to these duty station functional parameters.
- Implement strategies to protect the health and safety of the staff, patients, and their families with education. Involve community partners to facilitate resumption of this care. Prepare for an increase in mental health visits related to the isolation and attempts to accept the COVID-19 pandemic by the population.
- Develop non-acute COVID-19 primary and specialty care telehealth, identifying those in patients who may not have the means to participate. Involve community partners to facilitate this essential service.

Time is of the essence to continue the pandemic disaster cycle with a comprehensive post-COVID-19 health care delivery system RECOVERY analysis, plan, and operation at the local, regional, and state levels.
